# Surgical and oncological outcome after cytoreductive surgery and hyperthermic intraperitoneal chemotherapy for peritoneal mesothelioma: A retrospective single center experience

**DOI:** 10.1007/s00508-024-02460-z

**Published:** 2024-10-25

**Authors:** Catharina Müller, Michael Bergmann, Anton Stift, Thomas Bachleitner-Hofmann, Stefan Riss

**Affiliations:** Department of Surgery, Division of General Surgery, Comprehensive Cancer Center Vienna, Medical University of Vienna, Spitalgasse 23, 1090 Vienna, Austria

**Keywords:** Malignant peritoneal mesothelioma, Surgery, Peritoneal surface malignancies, Peritoneal mesothelioma, Oncology

## Abstract

**Background:**

Peritoneal mesothelioma (PM) is a rare disease with various histopathological subtypes. For malignant peritoneal mesothelioma and borderline subgroups locoregional therapy with cytoreductive surgery (CRS) and hyperthermic intraperitoneal chemotherapy (HIPEC) has been implemented. The aim of our study was to retrospectively present the outcome after CRS and HIPEC for patients with different subtypes of peritoneal mesothelioma.

**Methods:**

In total 15 patients received CRS and HIPEC due to peritoneal mesothelioma at our tertiary referral hospital between 2013 and 2022. Surgical and oncologic outcomes of 14 of those patients were retrospectively evaluated as one patient was lost to follow-up.

**Results:**

The cohort consisted of 9 patients with diffuse malignant peritoneal mesothelioma (64.3%), 3 patients with multicystic peritoneal mesothelioma (21.4%) and 2 patients with well-differentiated peritoneal mesothelioma (14.3%). Complete cytoreduction was possible in 85.7% (*n* = 12). The major complication rate was 28.6% (*n* = 4) and the reoperation rate was 14.3% (*n* = 2). Median follow-up was 55 months (standard error, SE 15.0%, 95% confidence interval, CI 25.6–84.4 months). Over this time period 64.3% (*n* = 9) had no evidence of disease, 21.4% (*n* = 3) were alive with disease and 14.3% (*n* = 2) died of peritoneal mesothelioma. The median recurrence-free survival of patients was 13 months (SE 13.0%, 95% CI 0.0–32.2 months). None of the patients with multicystic peritoneal mesothelioma had evidence of disease at the time of last follow-up.

**Conclusion:**

Patients with peritoneal mesothelioma should receive locoregional treatment as good oncological results can be achieved with reasonable postoperative morbidity. Thus, awareness is necessary for this rare but potentially aggressive disease to offer the best medical care.

## Introduction

Peritoneal mesothelioma (PM) is a rare disease originating from the serosal layer of the peritoneum. Various subtypes can be distinguished histologically that vary according to their malignant potential [1]. While diffuse malignant peritoneal mesothelioma (DMPM) is an aggressive malignant disease with invasive growth and high mortality, multicystic peritoneal mesothelioma (MCPM) and well-differentiated papillary peritoneal mesothelioma (WDPM) are tumors with low malignant potential, thus classified as borderline. Nevertheless, MCPM has a high risk of recurrence and WDPM is known for the risk of malignant transformation [2, 3]. Diffuse malignant peritoneal mesothelioma can further be subclassified in epithelioid, sarcomatoid and biphasic subtype. Not only the malignant potential but also the incidence and etiology vary between subtypes of peritoneal mesothelioma. The incidence rate for peritoneal mesothelioma is 0.5–3 cases per million, only 3–5% of peritoneal mesotheliomas are MCPM and WDPM occurs even less frequently [2, 4, 5]. In the histological subgroups of DMPM the epithelioid subtype is the most frequent while the sarcomatoid subtype is the most aggressive subtype. The DMPM is associated with asbestos exposure similar to pleural mesothelioma and is equally distributed between the sexes, whereas MCPM and WDPM mainly affect young women with no prior history of asbestos exposure [5, 6]. The MCPM and WDPM are associated with abdominal inflammation, previous surgery and endometriosis.

As PM primarily only affects the abdominal cavity and systemic treatment is often insufficient, locoregional treatment with cytoreductive surgery (CRS) and hyperthermic intraperitoneal chemotherapy (HIPEC) was implemented for the treatment of mesothelioma. It could be shown that the outcome for DMPM significantly depends on the treatment applied with CRS and HIPEC having the best long-term outcome results [7, 8]; however, a high number of patients are unresectable at the time of diagnosis and do not qualify for primary resection. Due to the low incidence, treatment strategies especially for peritoneal mesothelioma with low malignant potential, such as multicystic peritoneal mesothelioma and well-differentiated papillary mesothelioma, are based on retrospective cohort studies or case series as no randomized phase III studies are available. The recent guidelines from the peritoneal surface oncology group international (PSOGI) recommend cytoreductive surgery (CRS) and hyperthermic intraperitoneal chemotherapy (HIPEC) for all forms of peritoneal mesothelioma [6]. We present the outcome of patients with peritoneal mesothelioma undergoing surgical cytoreduction and intraperitoneal chemotherapy as a single center experience.

## Material and methods

We retrospectively analyzed the outcome of patients with peritoneal mesothelioma who received cytoreductive surgery (CRS) and hyperthermic intraperitoneal chemotherapy (HIPEC) at our tertiary referral center from 2013 to 2022. The study was approved by the Institutional Review Board (1771/2017).

**Fig. 1:**
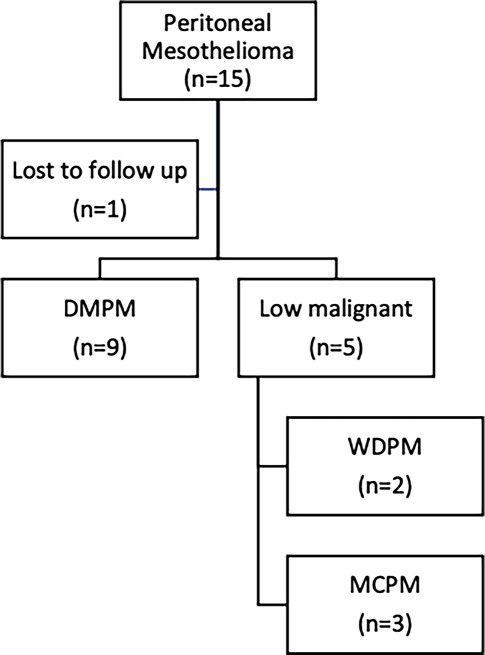
Figure 1 shows a flow diagram of patientsʼ inclusion

We identified 15 patients with peritoneal mesothelioma. Data were collected from our clinical database and individual patient chart review. One patient was lost for follow up at 3 months postoperatively. Thus, we finally included 14 patients with peritoneal mesothelioma in our study (Fig. 1).

The collected parameters included preoperative clinical data, perioperative details and postoperative outcome parameters. Baseline characteristics and outcome data consisted of age, sex, body mass index (BMI), American Society of Anesthesiologists classification (ASA), application of systemic chemotherapy and prior tumor-related surgery. Furthermore, the peritoneal carcinomatosis index (PCI) was calculated at the time of surgery, completeness of cytoreduction score (CC) was noted at the end of resection [9]. The extent of surgical resection and length of surgery were documented. Postoperative complications were classified according to the Clavien-Dindo classification and major complications were defined as grade III–V [10].

All patients had surgical cytoreduction and intraperitoneal chemotherapy. The intent of surgical cytoreduction was to remove all visible tumor and included selective parietal and visceral peritonectomy according to the macroscopic tumor involvement. Omentectomy, cholecystectomy and appendectomy were performed routinely independent of disease involvement. Further resection depended on macroscopic tumor involvement.

The intraperitoneal chemotherapy protocol was standardized with closed application of chemotherapy for 60 min at 42 °C with one inflow drain in the pelvis and two outflow drains in the upper abdomen. The chemotherapy regimen consisted of cisplatin (75 mg/m^2^) and doxorubicin (15 mg/m^2^).

### Statistical analysis

Statistical analysis was performed with IBM Statistical Package for the Social Sciences (SPSS) version 27 for Mac (SPSS, Chicago, IL, USA). Descriptive data were represented as median and interquartile range (IQR) or numbers (*n*) and percentages accordingly. Due to the small sample size no further statistical testing for group differences was done. For outcome data for Kaplan Meier curve was calculated. Overall survival (OS) was counted from the date of surgery and time to recurrence or recurrence-free survival (RFS) was defined as the period from CRS and HIPEC to first detection of recurrent disease. If neither death nor recurrence occurred in the time of follow-up these patients were censored at the time of last visit. The OS and RFS were expressed as median with standard error (SE) and 95% confidence interval (CI).

## Results

### Baseline characteristics

The median age of our cohort was 56 years (IQR 38.5–66.0 years). The distribution of sexes was equal with 50% male (*n* = 7) and 50% female patients (*n* = 7). According to the ASA score 57.7% were relatively fit patients with a score of 1 or 2. None of the patients had a score higher than 3. The most frequent comorbidity was arterial hypertension (*n* = 8, 57.1%), two patients had pulmonary risk factors with one patient suffering from chronic obstructive pulmonary disease (COPD) (7.1%), one from bronchial asthma (7.1%) and two patients had non-insulin-dependent diabetes mellitus type II (14.3%). Median BMI was 26.5 (IQR 21.6–28.1). According to the BMI 57.1% (*n* = 8) of our patients were overweight and 3 patients had a BMI of greater than 30 (21.4%).

Out of the whole cohort 9 patients had DMPM (64.3%), 3 patients MCPM (21.4%) and 2 patients suffered from WDPM (14.3%). The cohort of DMPM could further be subclassified in 7 patients with epithelioid (77.8%) and 2 patients with biphasic subtype (22.2%). We had no patient with sarcomatoid subtype in our cohort as only patients finally qualifying for surgery were selected. None of the patients had lymph node involvement.

In total, six patients had previous tumor-related surgery (42.9%) all of them being diagnostic laparoscopy or laparotomy with biopsy, one patient had a concomitant omentectomy and one patient an adnexectomy.

Further patient baseline characteristics divided by subtypes in detail are outlined in Table 1.

**Table 1:** Baseline characteristics overall and for the subgroups of low malignant peritoneal mesothelioma (WDPM, MCPM) and diffuse malignant peritoneal mesothelioma (DMPM)

	Diffuse malignant peritoneal mesothelioma (DMPM), *n* = 9	Low malignant peritoneal mesothelioma (WDPM, MCPM), *n* = 5	Overall, (*n* = 14)
Age (years)	56.0 (44.5–68.5)	56.0 (35.0–61.0)	56 (38.5–66.0)
*Sex*			
Female	4 (44.4)	3 (60.0)	7 (50.0)
Male	5 (55.6)	2 (40.0)	7 (50.0)
BMI	22.4 (22.7–28.3)	26.6 (19.8–31.5)	26.5 (21.6–28.1)
*ASA*			
1	1 (11.1)	2 (40.0)	3 (21.4)
2	4 (44.4)	1 (20.0)	5 (35.7)
3	4 (44.4)	2 (40.0)	6 (42.9)
*Previous tumor-related surgery*			
Yes	2 (22.2)	4 (80.0)	6 (42.9)
No	7 (77.8)	1 (20.0)	8 (57.1)
*Systemic chemotherapy*			
Yes	9 (100.0)	1 (20.0)	10 (71.4)
No	0 (0.0)	4 (80.0)	4 (28.6)
*Neoadjuvant chemotherapy*			
Yes	6 (66.7)	0 (0.0)	6 (42.9)
No	3 (33.3)	5 (100.0)	8 (57.1)
PCI	20 (18–29)	8 (8–10)	18 (8–24)
*PCI* *>* *17*			
Yes	8 (88.9)	0 (0.0)	8 (57.1)
No	1 (11.1)	4 (80.0)	5 (35.7)
Unknown	0 (0.0)	1 (20.0)	1 (7.1)
*CC*			
0	7 (77.8)	5 (100.0)	12 (85.7)
2	2 (22.2)	0 (0.0)	2 (14.3)
Length of operation (min)	545 (488–657)	415 (335–542)	518 (453–616)
*Postoperative complication*			
Yes	5 (55.6)	2 (40.0)	7 (50.0)
No	4 (44.4)	3 (60.0)	7 (50.0)
*Major postoperative complication*			
Yes	3 (33.3)	1 (20.0)	4 (28.6)
No	6 (66.7)	4 (80.0)	10 (71.4)
*Adjuvant chemotherapy*			
Yes	5 (55.6)	1 (20.0)	6 (42.9)
No	4 (44.4)	4 (80.0)	8 (57.1)

Data are represented in median (IQR) or numbers (percentages)

*CC 0* is complete,* CC 2* incomplete cytoreduction

*ASA* American Society of Anesthesiologists classification, *BMI* body mass index, *PCI* Peritoneal Cancer Index, *CC* Completeness of Cytoreduction, *WDPM* well-differentiated papillary peritoneal mesothelioma, *MCPM* multicystic peritoneal mesothelioma

### Surgical data and outcome

The median PCI in our cohort was 18 (IQR 8–24). The median operation time was 518 min (IQR 453–616 min). Patients with MCPM and WDPM had a median PCI of 8 (IQR 8–10) and patients with malignant peritoneal mesothelioma had a median PCI of 20 IQR (IQR 18–29). Accordingly, the median operation time was 415 min (IQR 335–542 min) for low malignant subgroups and median 545 min (IQR 488–657 min) duration of the surgery for malignant peritoneal mesothelioma.

In addition to routine peritonectomy, omentectomy, appendectomy and cholecystectomy hysterectomy and bilateral salpingo-oophorectomy were performed in 4 patients (28.6%), low anterior rectum resection in 4 patients (28.6%), colon resection in 3 patients (21.4%), splenectomy in 5 patients (35.7%), small bowel resection in 1 patient (7.1%) and atypical gastric resection in 1 patient (7.1%). Overall, 12 patients (85.7%) had complete cytoreduction (CC 0) and 2 patients (14.3%) had incomplete cytoreduction (CC 2). Complete tumor removal was unsuccessful due to high tumor load with a PCI of 33 in both patients suffering from DMPM as underlying disease. Accordingly, only omentectomy was performed and HIPEC was applied to possibly reduce the production of malignant ascites [2].

Patients stayed a median of 1 day in the intensive care unit (ICU) postoperatively (IQR 1–2 days). The length of hospital stay was a median of 11 days (IQR 9–14 days). The overall major complication rate in our cohort was 28.6% (*n* = 4). In detail, two patients needed additional surgery due to wound infections, one patient had pleural puncture due to pleural effusion and one patient had radiologically guided drainage due to deep surgical site infection with an intra-abdominal retention. Accordingly, the reoperation rate was 14.3% (*n* = 2). Despite the intraperitoneal use of cisplatin only one patient had renal impairment postoperatively and temporarily needed hemodialysis. The 90-day mortality rate was 0.0%.

### Oncologic outcome

Apart from CRS and HIPEC 71.4% (*n* = 10) of the patients also received systemic chemotherapy: one patient received carboplatin/pemetrexed and 9 patients received cisplatin/pemetrexed. Out of the cohort of patients with DMPM 6 (66.7%) were borderline resectable and therefore received neoadjuvant chemotherapy. At the time of surgery patients still had a PCI of greater than 18.

**Fig. 2:**
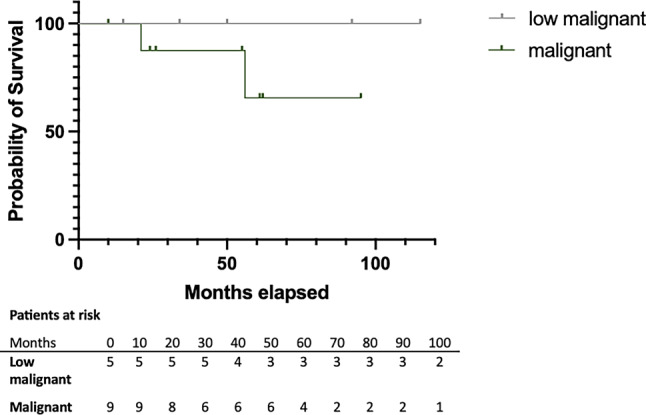
Survival curves of patients with mesothelioma with low malignant potential (MCPM, WDPM) and diffuse malignant peritoneal mesothelioma

Median time of follow-up was 55 months (SE 15.0%, 95% CI 25.6–84.4 months) and 6 patients (42.9%) had a local recurrence. Median time to recurrence was 13 months (SE 13.0%, 95% CI 0.0–32.2 months). The overall 2‑year RFS was 67.7% and 5‑year RFS 38.7%. Two patients with a recurrence were treated with repeated surgery and both were without evidence of disease at the last date of follow-up. Thus, at the time of last analysis 64.3% (*n* = 9) had no evidence of disease, 21.4% (*n* = 3) were alive with disease and 14.3% (*n* = 2) died of the peritoneal mesothelioma (Table 2). In our small cohort, the overall 2‑year survival was 91.6% and the 5‑year survival rate of 76.4%. Survival curves are represented in Fig. 2 for patients with mesothelioma with low malignant (MCPM, WDPM) and high malignant potential (DMPM) separately.

**Table 2:** Outcome data for different histological subtypes of mesothelioma patients

	AWD, *n* (%)	DOD, *n* (%)	NED, *n* (%)
Overall	3 (21.4)	2 (14.3)	9 (64.3)
Benign multicystic mesothelioma	0 (0.0)	0 (0.0)	3 (100)
Diffuse malignant peritoneal mesothelioma	2 (22.2)	2 (22.2)	5 (66.6)
Well-differentiated papillary mesothelioma	1 (50.0)	0 (0.0)	1 (50.0)

*AWD* alive with disease, *DOD* dead of disease, *NED* no evidence of disease

According to histological subtypes, all patients with multicystic peritoneal mesothelioma (*n* = 3) had no evidence of disease at the time of follow-up (median follow-up 50 months). Of these patients 1 had recurrence after 13 months and had another CRS with complete cytoreduction. Out of the two patients with well-differentiated papillary peritoneal mesothelioma one had no evidence of disease after a follow-up of more than 9 years (115 months) and the second one also had pleural involvement and was alive with disease after 15 months under immunotherapy with the first recurrence after an interval of 8 months. In the cohort of patients with DMPM 5 patients (55.6%) had no evidence of disease, 2 patients (22.2%) were alive with disease and 2 patients (22.2%) died of the disease. Interestingly, one patient with incomplete cytoreduction had complete radiological response with systemic chemotherapy with cisplatin and pemetrexed.

## Discussion

In our study we found good oncological outcome for peritoneal mesothelioma in patients who received locoregional treatment with surgical removal of the tumor combined with heated intraperitoneal chemotherapy. Over a time period of 10 years, we treated 14 patients suffering from peritoneal mesothelioma with CRS in combination with HIPEC. The procedures were distributed equally over time so that 1–2 patients with this rare disease were treated surgically at our tertiary referral center per year. The main histological subtype was diffuse peritoneal mesothelioma with 64.3% followed by multicystic peritoneal mesothelioma (21.4%) and well-differentiated papillary mesothelioma (14.3%).

Before local treatment with radical surgery and heated intraperitoneal chemotherapy was established, the median OS of patients with malignant peritoneal mesothelioma was about 1 year [11]. With the implementation of CRS and HIPEC as a treatment option the survival was significantly prolonged to about 30–90 months [8, 11–13]. Our survival data are compatible with previous literature; however, it has to be mentioned that we included DMPM, WDPM and MCPM in the overall survival calculation.

In should be noted that we operated patients with a relatively high PCI especially in the DMPM subgroup, even though guidelines to date recommend PCI > 17 as a relative contraindication and high-risk feature [6]. The 5‑year survival rate was 76.4%. Thus, PCI should probably not be given priority in the selection of patients for surgery. Even though PCI correlates with survival [14], one of the largest multi-institutional data registries that provided the basis for the recommendation of primary CRS in combination with HIPEC for patients suffering from resectable DMPM showed that CRS and HIPEC was generally also performed for a more extensive tumor load [8]. Yan et al. reported a median PCI of 20 [8]. Recently long-term outcome of patients with malignant peritoneal mesothelioma of a single high-volume center were published that showed an OS of 3.3 years in a cohort of patients with a mean PCI of 18.7. Surprisingly, the outcome for malignant peritoneal mesothelioma was significantly better than in patients with peritoneal metastasized colorectal cancer. Moreover, patients seemed to profit from iterative CRS and HIPEC for disease relapse with a median OS for patients with a second CRS and HIPEC of 67.7 months [15]. In our cohort we found two patients who received surgery for recurrence of disease. Both were without evidence of disease at the date of last follow-up.

Patient case selection, however, is essential not only for the best oncological outcome but also to prevent unnecessary surgery for patients. Prognostic factors for survival in DMPM are the histological subtype, Ki67 status, lymph node involvement, extent of disease and completeness of cytoreduction [8, 16, 17]. In cases of high risk features systemic chemotherapy before or after surgery are indicated. In our cohort none of the patients had lymph node involvement, only two patients had biphasic malignant mesothelioma (14.3%) and none of the patients had a sarcomatoid subtype.

The high PCI of our patients may explain the high rate of systemic chemotherapy used in the cohort. Even though upfront surgery is recommended in the case of resectable disease, we treated patients with borderline situations who received preoperative systemic chemotherapy. To date the standard treatment for advanced unresectable and borderline resectable malignant peritoneal mesothelioma is cisplatin and pemetrexed, which is based on a phase III clinical trial for pleural mesothelioma [18]; however, the effect of these agents was also shown in malignant peritoneal mesothelioma [19–21]. All patients with preoperative neoadjuvant chemotherapy still had a high intraoperative PCI indirectly indicating poor response to systemic chemotherapy in our cohort. In contrast, one patient with incomplete cytoreduction had complete radiological response with postoperative systemic chemotherapy. The relatively poor effect of chemotherapy for peritoneal mesothelioma is well known and therefore surgical cytoreduction is first line in resectable patients. Recently, new treatment options such as immunotherapy have been the subject of clinical studies for malignant peritoneal mesothelioma. As mentioned above peritoneal mesothelioma is a rare disease and treatment concepts are often initially based on findings made in pleural mesothelioma patients. For advanced unresectable pleural mesothelioma the combination of ipilimumab, anti-CTLA 4 (cytotoxic T-lymphocyte antigen 4) antibody) and nivolumab, anti-PD-1 (programmed cell death protein 1) antibody, has become the first-line treatment based on the clinical phase III trial CheckMate 743 [22]. In this trial Baas et al. also found that a significant survival benefit for patients with pleural mesothelioma was especially shown in the histological subgroup of patients suffering from non-epithelioid mesothelioma [22]. The majority of patients suffering from malignant peritoneal mesothelioma have an epithelioid subtype. Nevertheless, for peritoneal involvement a cohort study of 29 patients showed an OS of 19 months when immune checkpoint inhibitors were administered for advanced DMPM [23]. In this study the combination of nivolumab with ipilimumab was the mainly used regimen followed by single agent treatment with nivolumab, atezolizumab and pembrolizumab. The objective response rate was 19.2% [23]. The combination of atezolizumab (anti-PD-L1) and bevacicumab (anti-VEGF) showed a 40% response in advanced peritoneal mesothelioma [24]. A retrospective case series using pembrolizumab (anti-PD1) reported on patients with progression-free survival of more than 2 years [25]. Even though few data are available for malignant peritoneal mesothelioma, immunotherapy is recommended as a systemic therapy for unresectable peritoneal mesothelioma in current clinical guidelines for peritoneal mesothelioma [26]. Immunotherapy has not explicitly been implemented in the neoadjuvant setting but new forms of systemic treatment may lead to a higher number of patients converting from borderline resectable to operable disease in the future.

High PCI and high rate of systemic chemotherapy applied are an indirect sign for a high-risk population in our cohort. The two patients with the highest PCI levels were those with incomplete cytoreduction (CC 2). HIPEC was applied in line with recent guidelines recommending that intraperitoneal chemotherapy should be considered if the remaining disease burden is limited after debulking [6]; however, the question of applying HIPEC after incomplete CRS is still controversial.

In the neoadjuvant setting the application of intraperitoneal chemotherapy was able to reduce ascites production and even pain and may eventually increase the resection rate [27]. In the adjuvant setting early postoperative intraperitoneal chemotherapy (EPIC) and long-term normothermic intraperitoneal chemotherapy (NIPEC) are discussed after CRS and HIPEC. Sugarbaker et al. showed that NIPEC that consists of long-term intraperitoneal chemotherapy over 6 cycles through an intraperitoneal port that is placed during the CRS and HIPEC procedure leads to significantly longer survival rates whereas EPIC in comparison to CRS and HIPEC alone has no significant survival benefit [28].

In the literature few data can be found for the use of pressurized intraperitoneal aerosol chemotherapy (PIPAC) in malignant peritoneal mesothelioma, where chemotherapy is administered directly to the abdominal cavity in the form of aerosols under high pressure with a laparoscopic approach. In a retrospective cohort study of 29 patients with advanced malignant peritoneal mesothelioma PIPAC led to histological tumor regression in > 50% and a median OS of 26 months with better quality of life and a trend to reduction of ascites [29]. These findings were similar in another cohort study where 55% of patients with initially non-resectable disease could be converted to undergo surgical cytoreduction and HIPEC [30]. Patients with secondary CRS and HIPEC then had a better oncological outcome with significantly longer progression-free survival [30].

All patients with borderline forms of peritoneal mesothelioma were alive at a median follow-up of about 4 years and only one had evidence of disease. In accordance with the literature all patients with MCPM had no evidence of disease during follow-up even though recurrent cytoreduction was necessary [31, 32].

The limitations of our study are the design being a retrospective data analysis and the low case load that is attributed to the rarity of disease but also to the single center analysis. The low case load may also be due to the missing presentation of patients with peritoneal mesothelioma at a specialized center. Furthermore, the number of patients actually receiving CRS and HIPEC is further decreased due to the high rate of unresectable disease at the time of presentation mainly due to the delayed diagnosis because of unspecific clinical symptoms. Thus, treatment options to achieve a higher rate of secondary resectable disease is essential. This can be achieved by different administration forms of intraperitoneal chemotherapy or new systemic substances, such as immunotherapeutic agents.

## Conclusion

Cytoreductive surgery in combination with heated intraperitoneal chemotherapy in patients with peritoneal mesothelioma resulted in a 2-year survival rate of 91.6%. Thus, awareness has to be created for this rare disease so that patients can benefit from available surgical treatment options.

## Data Availability

The raw data supporting the conclusions of this article will be made available by the authors on request.
